# Immune-nutritional interactions in endometriosis: current evidence and future perspectives in disease management

**DOI:** 10.3389/fimmu.2026.1940772

**Published:** 2026-09-07

**Authors:** Julia Duraj, Klara Kowalczyk, Paulina Żelechowska, Justyna Agier

**Affiliations:** 1 Department of Molecular Neurochemistry, Medical University of Lodz, Lodz, Poland; 2 International Doctoral School, Medical University of Lodz, Lodz, Poland; 3 Department of Microbiology, Genetics and Experimental Immunology, Centre of Molecular Studies on Civilization Diseases MOLecoLAB, Medical University of Lodz, Lodz, Poland

**Keywords:** diet, dysmenorrhea, endometriosis, immune dysregulation, immunonutrition, inflammation, nutrition therapy, pelvic pain

## Abstract

Endometriosis is a chronic estrogen-dependent inflammatory disorder affecting approximately 10% of women of reproductive age. It remains a major contributor to chronic pelvic pain, infertility, and reduced quality of life. Despite considerable advances in elucidating its genetic, hormonal, and immunological basis, current therapeutic strategies frequently provide incomplete symptom relief, may be associated with adverse effects, and fail to prevent disease recurrence. Therefore, increasing attention has been directed toward modifiable environmental factors, particularly nutrition, as supplementary strategies for disease management. This review comprehensively examines the evidence connecting dietary patterns and bioactive food components with the immunopathogenesis of endometriosis. It explores the potential role of nutrition in modulating key biological processes involved in endometriosis pathophysiology and progression, including immune dysregulation, chronic inflammation, oxidative stress, angiogenesis, estrogen metabolism, and gut microbiota imbalance. Particular emphasis is placed on the Mediterranean diet and selected dietary components, such as omega-3 fatty acids, vitamin D, resveratrol, epigallocatechin-3-gallate, and probiotics, as well as dietary interventions like low-FODMAP and gluten-free diets. The findings are discussed in relation to mechanistic evidence and emerging observations. Substantial clinical data supporting specific dietary interventions are scarce, and most dietary guidelines are based on preliminary research. Future investigations that incorporate nutritional interventions alongside immune profiling, microbiome analysis, and multi-omics technologies may facilitate the development of individualized nutritional strategies as adjuncts within the framework of precision medicine for women affected by endometriosis.

## 1 Introduction

Endometriosis constitutes a chronic inflammatory gynecological condition distinguished by the proliferation of tissue resembling the endometrium, which typically lines the uterus, occurring beyond its customary location (1–3). The etiology and pathology of disease have persisted as subjects of scholarly debate since the publication of the initial comprehensive pathological description in 1860 (3). While Dr. Karel Freiherr von Rokytanský, an Austrian physician of Czech heritage, is acknowledged as a pioneer in endometriosis research owing to his detailed, comprehensive descriptions of the histological and morphological features of the condition, it took over thirty years for a hypothesis concerning its origin to be developed. This undertaking was executed by the Berlin pathologist Friedrich von Recklinghausen, who published extensive descriptions of a series of endometriotic nodules, suggesting that they originated from Wolffian bodies (4). In 1927, John A. Sampson proposed two mechanisms that could account for the dissemination and implantation of endometrial tissue: hematogenous dissemination through the venous circulation, leading to metastatic or embolic endometriosis (5), and retrograde menstrual flow via the fallopian tubes, resulting in the implantation of endometrial tissue within the peritoneal cavity (6). In 1940, he further developed the latter mechanism into the implantation theory, suggesting that the ectopic development of endometrial tissue originates from the migration of mucosal fragments during menstruation through the fallopian tubes into the peritoneal cavity (7). This concept remains one of the most widely accepted hypotheses concerning the pathogenesis of endometriosis. An alternative theory implicates metaplasia, which denotes the capacity of undifferentiated peritoneal cells to metamorphose into cells characteristic of the uterine mucosa (8). Currently, it is recognized that endometriosis has a complex, multifactorial nature. Ectopic, mislocalized endometrial-like tissue may occur in various body regions, with the ovaries, fallopian tubes, and pelvic organs representing the most affected areas. Although the precise etiology of endometriosis remains elusive, a substantial body of research indicates that genetic predisposition constitutes a major factor contributing to the onset and progression of this condition (2, 9). Furthermore, certain studies have established a correlation between inflammatory mechanisms, oxidative stress, hormonal dysregulation, and the development of endothelial dysfunction, all of which may influence disease progression (9, 10).

Approximately ten percent of women in their reproductive years, equivalent to one hundred ninety million women worldwide, are affected by endometriosis (1). The prevalence of the condition is most notable among women aged 25 to 35 and demonstrates variability across various ethnic groups (11). In the meta-analysis conducted by Bougie et al. (12), the authors demonstrated, based on an examination of 18 studies, that Black women exhibited a lower rate of endometriosis diagnosis in comparison to White women. Conversely, Asian women presented a higher rate of diagnosis. Parazzini et al. (13) conducted a similar meta-analysis, which noted that the prevalence of endometriosis was distributed as follows: 1.4% among European women, increasing to 5.7% in studies conducted in the United States, and reaching 15.4% in studies conducted in Asia. A significant gap persists in the understanding of endometriosis regarding the impact of socioeconomic factors on its prevalence. Nonetheless, researchers in the field of endometriosis have concluded that, alongside age and race, variables such as body mass index (BMI), and lifestyle-related behaviors, including alcohol consumption and cigarette smoking, may also be associated with the occurrence of endometriosis (14).

Endometriosis is characterized by the occurrence of chronic pelvic pain associated with an imbalance between sensory and sympathetic innervation (15). Women diagnosed with endometriosis are statistically more likely to experience menstrual symptoms, such as severe pelvic pain, heavy or prolonged menstrual bleeding, pain associated with ovulation, mental health complications, and disturbances related to both bowel and urinary functions, in comparison to individuals who do not suffer from this condition (16). The disease significantly impacts daily life by affecting physical activities, social interactions, and emotional well-being (15). Furthermore, women diagnosed with endometriosis encounter challenges in conceiving, which may arise from anatomical distortions and inflammation-induced alterations in the pelvic and endometrial microenvironment (17, 18). A comprehensive understanding of the molecular mechanisms underlying these symptoms is paramount for enhancing the diagnosis and treatment of endometriosis. Despite its widespread prevalence, diagnosing endometriosis remains challenging. Over the years, laparoscopy with histological confirmation has been considered the gold standard (19). Recent advancements in imaging techniques, notably transvaginal and transrectal ultrasound, have significantly improved the detection of endometriotic cysts and deep infiltrating lesions. Laboratory markers have demonstrated restricted utility in diagnostic processes, as evidenced by the serum CA-125 (cancer antigen-125) level, which is predominantly elevated in advanced stages (20). However, the latest findings suggest that increased endometrial *FUT4* mRNA expression may serve as a more specific marker of this condition (21). The diagnostic procedure typically encompasses a comprehensive clinical evaluation, laboratory assessments, imaging techniques, and surgical staging, complemented by histological analysis (22). Endometriosis treatment strategies involve both medical and surgical approaches. The first-line options for managing symptoms include the combined use of hormonal contraceptives, with or without nonsteroidal anti-inflammatory drugs. A referral to a gynecologist for surgical management is justified if empirical therapy fails or if women express a desire for pregnancy but encounter difficulties conceiving (23, 24).

The contemporary medical paradigm asserts that nutritional assessment should be incorporated as a fundamental component of the patient’s comprehensive health evaluation. It enables the comparison of the patient’s clinical condition with the selected nutritional model and substantiates the implementation of modifications to enhance their well-being. Dietary interventions may be advantageous in managing endometriosis and, importantly, in alleviating associated pain. The potential importance of nutrition in endometriosis is indicated by the influence of dietary components on estrogenic activity and inflammatory mechanisms. This review explores the immunological foundations of endometriosis, emphasizing how chronic inflammation and immune dysregulation contribute to the disease’s progression. It provides a comprehensive analysis of the existing evidence on the effects of dietary interventions, particularly those involving anti-inflammatory and immunomodulatory diets, in managing endometriosis-related symptoms. Furthermore, the article offers an extensive examination of immune-related pathways, which could serve as the basis for developing targeted therapies grounded in dietary interventions.

A comprehensive literature review was conducted utilizing the PubMed database, employing various combinations of keywords “endometriosis”, “nutrition”, “inflammation”, and “immune dysregulation”. The investigation further included specific immune cell populations and inflammatory mediators involved in the pathogenesis of endometriosis. The search encompassed publications from 2021 to June 2026. Studies exploring the relationships between dietary factors, inflammation, and endometriosis were incorporated, covering cell culture research, *in vivo* animal studies, and clinical trials assessing dietary interventions in women with endometriosis. Duplicate publications, studies with inconclusive results, articles with inaccessible full texts, and publications outside the scope of this review were excluded. Following the screening and selection process, a total of 56 publications directly relevant to the scope of this review were included.

## 2 Risk and protective factors in endometriosis

**Figure 1:**
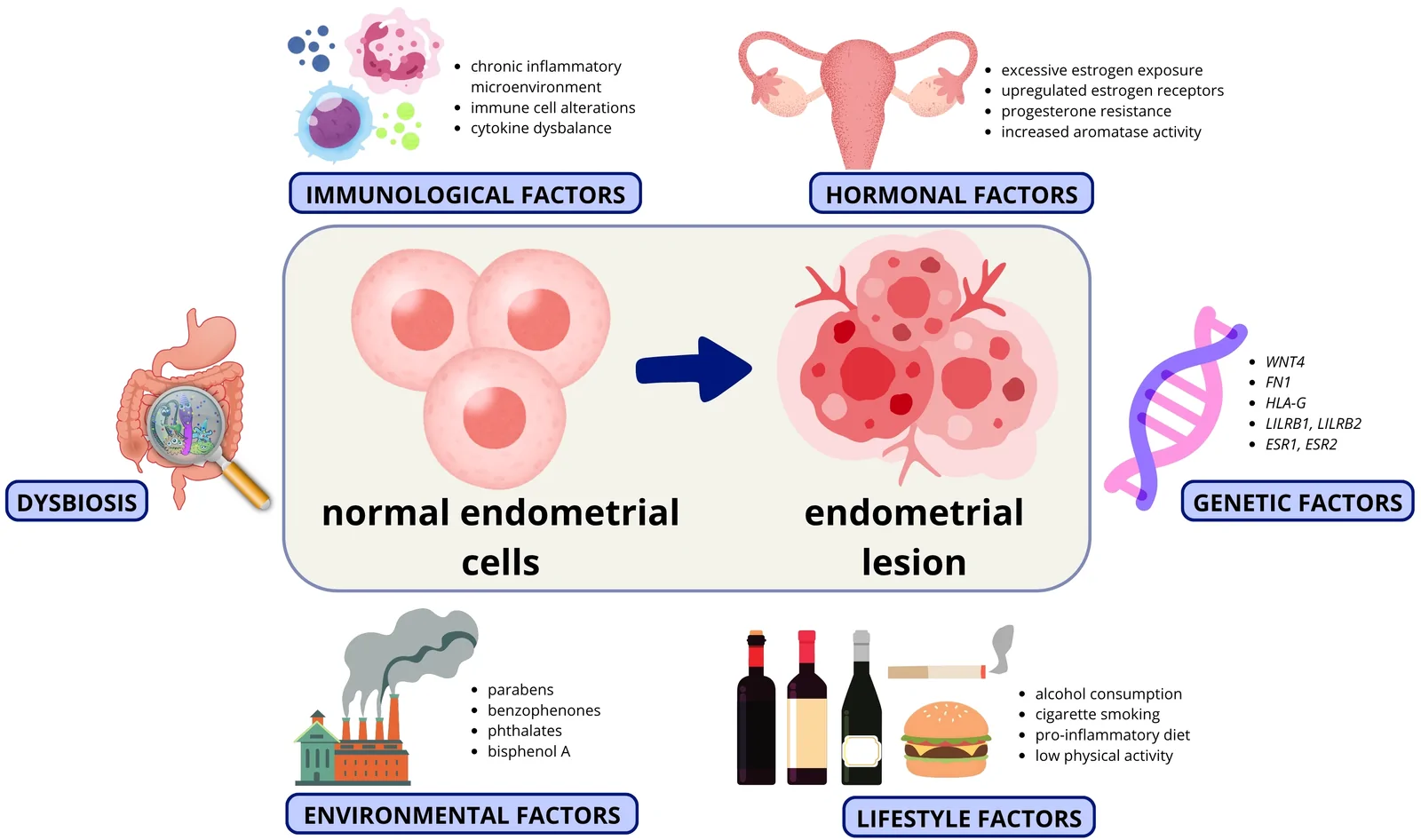
Potential factors contributing to the development of endometriosis.

The development of endometriosis is influenced by non-modifiable factors, such as genetic predisposition, as well as modifiable initiators, including environmental and lifestyle components. **Figure 1** provides a summary of the potential factors influencing the development of endometriosis identified to date.

### 2.1 Genetic factors

There has been a growing academic interest in investigating the influence of genetic factors in the etiology of endometriosis, emphasizing their importance as a crucial determinant in the progression of the condition (2). Examinations employing familial aggregation, twin analysis, and comprehensive genome-wide association studies (GWAS) have conclusively validated the heritability of this condition (25, 26). Epidemiological research indicates that up to 50% of individuals diagnosed with endometriosis possess a hereditary component (2), while the remaining cases are attributable to multifactorial non-genetic influences (2, 27). Nonetheless, studies propose that approximately 26% of the risk associated with endometriosis can be attributed to genetic variants influencing fundamental biological processes (28). These processes encompass tissue remodeling, cellular proliferation, and immune system functionality, all of which are essential for the establishment and maintenance of ectopic endometrial tissue. Comprehending how specific genetic factors influence these mechanisms offers valuable insights into the fundamental pathophysiology of the disease.

The formation and differentiation of numerous cell types during both embryonic development and adult homeostasis depend on the WNT family member 4 (*WNT4*) gene (29). This gene has been associated with the development of female reproductive tissues, including the uterus and ovaries (30), and certain polymorphisms in this gene are acknowledged as contributing to the pathogenesis of endometriosis (31). *WNT4* constitutes a significant factor in the progression of the condition, particularly in its capacity to regulate cellular proliferation beyond the uterus (31, 32). In patients experiencing infertility, endometriosis is associated with strictly defined single-nucleotide polymorphisms (SNPs) on *WNT4* gene (33). Variants of this gene may interfere with normal tissue development, potentially leading to the formation of ectopic endometrial lesions (34).

The *FN1* gene, which encodes the extracellular matrix glycoprotein fibronectin, plays a crucial role in cellular adhesion and migration. The balance between extracellular matrix production and degradation is essential for maintaining proper tissue function. The study conducted by Sapkota et al. (35) demonstrated a strong association between a locus within or near the *FN1* gene and endometriosis, particularly with moderate-to-severe forms of the condition. The level of *FN1* expression is elevated in the eutopic endometrial tissue samples of women compared to healthy controls (36), thereby promoting the adhesion of ectopic tissue to adjacent structures. This may contribute to the persistence and viability of lesions, thus facilitating their ongoing growth and the manifestation of symptoms associated with endometriosis. In the analysis conducted by Matalliotaki et al. (37), involving 166 women diagnosed with endometriosis, a genetic association between the *FN1* SNP and the condition was identified at both the genotypic and allelic levels.

In addition to genes related to tissue development, an equally important gene involved in the progression of endometriosis is *HLA-G* (Human Leukocyte Antigen-G), which has been shown to regulate immune tolerance. Advanced stages of the disease are associated with elevated levels of the protein encoded by this gene (38). Polymorphisms in the *HLA-G* gene have been demonstrated to influence the host immune response to ectopic endometrial tissue – a pivotal element in the pathogenesis of endometriosis (39). The immune system’s ability to identify and eradicate abnormal tissue is vital for preventing the establishment and progression of ectopic endometrial cells. The atypical expression of the *HLA-G* gene in endometriotic lesions suggests that mechanisms analogous to those activated during pregnancy, particularly localized immune tolerance, may facilitate the survival, implantation, and proliferation of endometrial cells beyond the uterine cavity. Moreover, specific *HLA-G* polymorphisms have been linked to a reduced susceptibility to endometriosis, highlighting the gene’s functional importance in regulating immune-mediated aspects of disease development. HLA-G may contribute to the body’s diminished capacity to effectively identify and eliminate ectopic endometrial tissue (40), thereby supporting its persistence, which can result in chronic inflammation, pain, and other clinical manifestations associated with endometriosis.

As well as *HLA-G*, other immune-related genes, such as *LILRB1* (Leukocyte Immunoglobulin-like Receptor B1) and *LILRB2*, also contribute to endometriosis susceptibility. Both are essential for regulating immune cell functions, and genetic variations within them may affect the immune system’s interactions with ectopic endometrial tissue. Bylińska et al. (39) demonstrated that the *LILRB1* rs41308748:AA genotype and the *LILRB2* rs383369:AG genotype are associated with increased susceptibility to the disease and its progression.

### 2.2 Environmental and lifestyle factors

Although genetic factors appear to have a predominant influence on the progression of endometriosis, various environmental and lifestyle factors also contribute. Endocrine-disrupting chemicals (EDCs) are acknowledged as a class of exogenous chemical agents capable of interfering with the normal endocrine system function (41). This interference can disrupt hormonal balance and potentially trigger pathological processes related to estrogen-dependent conditions, such as endometriosis (42). EDCs have multiple entry routes into the human body, such as ingestion through food and water, inhalation of airborne particles and dust, and dermal absorption consequent to the use of cosmetics and personal care products (41, 42). This issue is particularly alarming considering that numerous widely utilized cosmetics include substances such as parabens and benzophenones, which are recognized to have endocrine-disrupting properties. Regular and frequent usage of these products can result in the continuous accumulation of these compounds within the human body, potentially leading to hormonal imbalance and heightened oxidative stress, both of which have been associated with the development and progression of endometriosis (43, 44).

In addition to chemical exposure, other modifiable lifestyle factors may contribute to the protective management of endometriosis. Epidemiological studies suggest that endometriosis is correlated with lower BMI and reduced levels of obesity, indicating an inverse relationship between BMI and the risk of endometriosis (45). The literature review by Bonocher et al. (46) documents that the evidence is inconclusive regarding the benefits of physical exercise as a risk factor for the disease, and no data exist concerning the potential impact of exercise on the progression of endometriosis. However, physical activity may affect the clinical manifestations of the condition by influencing hormonal regulation, specifically by reducing luteal estrogen levels and increasing sex hormone-binding globulin concentrations (47).

Consistent with prior findings, alcohol intake may promote the formation of reactive oxygen species (ROS), further supporting the oxidative stress hypothesis in endometriosis pathophysiology (48). Alcohol potentially influences endocrine signaling through interaction with pituitary-derived luteinizing hormone, which may result in an elevated secretion of estradiol by the ovaries (49). Given that endometriosis is an estrogen-driven condition, this mechanism may partially elucidate the observed correlations between alcohol intake and an elevated risk of the disease. It is imperative to acknowledge, however, that such effects are predominantly apparent at high levels of alcohol consumption.

Interestingly, reproductive practices such as breastfeeding have been recognized as protective factors (50). A meta-analysis demonstrated that breastfeeding was associated with a significantly reduced risk of endometriosis, although the underlying biological mechanisms remain to be fully elucidated.

## 3 Immunopathogenesis of endometriosis

An expanding body of evidence suggests that dysregulated immune responses could act as a precipitating factor primarily for deeply infiltrative endometriosis (DIE), a severe form of the disease distinguished by endometrial-like tissue that invades deeply into pelvic organs such as the bowel, bladder, and other structures. Researchers have recognized immune dysfunction as a critical factor in the initiation and progression of endometriosis, involving specific immune cells, including macrophages, neutrophils, natural killer (NK) cells, and dendritic cells (DCs). Furthermore, immune mediators are of substantial importance as they facilitate the functioning of cells that are essential for the survival, implantation, and dissemination of endometrial cells beyond the uterine cavity.

### 3.1 Cells

The immune landscape in endometriosis is significantly modified, especially within the peritoneal cavity, where immune cells exhibit phenotypic and functional deviations that facilitate the survival of ectopic endometrial cells. The body’s innate response to tissue injury or alteration is primarily governed by macrophages, which determine the most appropriate course of action to restore equilibrium. This includes, on one hand, the identification and eradication of pathogens, infected or transformed cells, and, on the other, the activation, proliferation, and differentiation of precursor or stem cells, as well as the formation of new blood vessels. Macrophages are situated near endometrial cells and play a crucial role in preserving homeostasis within the immune microenvironment (51). Macrophage abundance within the physiological endometrium varies throughout the menstrual cycle and attains its maximum during menstruation, a process vital for the effective removal of cellular debris in this period (52). It is postulated that endometriosis constitutes a macrophage-related pathology (53), as an increase in macrophage numbers is not detected within the eutopic endometrium of patients with the condition during a specific phase (54). Instead, an increase in macrophage recruitment has been observed in the endometrium of women with endometriosis, regardless of the hormonal cycle, and their ability to clear migrated endometrial fragments seems to be inefficient. Moreover, ectopic tissue in lesions exhibits an increased abundance of M2-polarized macrophages, which may contribute to angiogenesis (55, 56). Furthermore, macrophage recruitment toward nerve fibers may influence their development, which correlates with pain in patients with peritoneal endometriosis (57, 58). Emerging evidence suggests that dietary factors may modulate macrophage actions, thereby potentially influencing the inflammatory microenvironment of endometriotic lesions. In particular, vitamin D, short-chain fatty acids (SCFAs), dietary fiber, and polyphenols such as resveratrol have been shown to modulate macrophage phenotype and recruitment and inflammatory signaling through pathways involving nuclear factor kappa B (NF-κB), AMP-activated protein kinase (AMPK), signal transducer and activator of transcription (STAT)6, and epigenetic mechanisms (59–63). These findings provide a potential immunometabolic link between dietary exposures and macrophage-mediated inflammation.

Beyond their established roles in inflammation (64) and antimicrobial immunity (65, 66), mast cells (MCs) are now acknowledged as crucial regulators of the immune microenvironment and significant contributors to the pathogenesis of endometriosis (67). Over the past five years, there have been notable advances in the understanding of MC biology in this disease, recognizing them as central drivers of immune dysregulation, neuroinflammation, lesion progression, and pain development. MCs are integral components of the microenvironment associated with endometriosis and adenomyosis (68–70). They interact closely with macrophages, thereby contributing to a self-sustaining pro-inflammatory microenvironment (71), which is regulated by estrogen (72). Estrogen-activated MCs contribute directly to endometriosis-related pain *via* the GPR30-FGF2 signaling pathway (73). Ding et al. (74) demonstrated that MCs facilitate the progression of endometriosis by inducing epithelial-mesenchymal transition (EMT), and an increased infiltration of MCs correlates with a more invasive cellular phenotype. The researchers observed a significantly higher density of MCs in ectopic endometrium than in matched eutopic endometrium. Additionally, endometrial lesions were characterized by heightened expression of stem cell factor, a crucial regulator of MC recruitment, differentiation, and survival, thereby indicating a microenvironment favorable to MC accumulation and activation. The significance of MCs in the pathogenesis of endometriosis is predominantly attributable to their ability to produce a wide array of inflammatory, angiogenic, fibrogenic, and neuroactive mediators that influence the microenvironment of localized lesions. Velho et al. (75) demonstrated that histamine signaling is active in the lesion microenvironment, as evidenced by increased serum histamine concentrations in women with endometriosis compared to controls. Histamine released by MCs may stimulate receptors on sensory neurons, leading to sensitization, which is responsible for chronic pain (76). A study by Atiakshin et al. (69) also demonstrated the directed secretion of tryptase and carboxypeptidase A3 (CPA3) by MCs, suggesting their active role in remodeling the lesion microenvironment. Guo et al. (77) demonstrated that 17β-estradiol, through the estrogen receptor Erα, activates the NLRP3 inflammasome, leading to the subsequent activation of caspase-1 and an increase in the production of mature IL-1β.

An increasing body of evidence affirms the pivotal role of MCs in the progression of endometriosis and has consequently stimulated heightened interest in the development of therapeutic strategies targeting these cells. Presently, substantial research efforts are dedicated to identifying pharmacological approaches capable of preventing MC recruitment and activation, thereby mitigating chronic inflammation, lesion progression, fibrosis, neurogenesis, and associated pain in endometriosis. Yu et al. (78) demonstrated that salbutamol, a β2-adrenergic receptor (β2AR) agonist, significantly inhibits the progression of endometriosis in a murine model, with one of its key effects being a noteworthy reduction in MC infiltration within endometriotic lesions. Arangia et al. (79) showed in a rat model of endometriosis that fisetin, a natural polyphenol possessing anti-inflammatory and antioxidant properties, effectively restricts the development of endometriotic lesions, partly by inhibiting MC activity. Additionally, a study by Akarca-Dizakar et al. (80) revealed that coenzyme Q10 effectively limited the progression of endometriosis in a rat model, with one observed consequence being a reduction in MC infiltration within endometriotic tissue. Moreover, Genovese et al. (81) established in a murine model of endometriosis that cannabidiol significantly diminishes MC recruitment to the spinal cord, correlating with the attenuation of mechanisms responsible for pain sensitization. Nevertheless, the findings primarily derive from animal experimental models, and there exists a lack of clinical evidence to substantiate the efficacy of salbutamol, fisetin, coenzyme Q10, or cannabidiol in modulating MCs in women with endometriosis.

Neutrophils play a progressively prominent role in the advancement of endometriosis. These cells are highly versatile, executing functions such as surveillance, supporting angiogenesis, phagocytosis, antigen presentation, and cytokine production. Tariverdian et al. (82) observed that the frequency of neutrophils is elevated across all stages of endometriosis relative to women without the condition, with statistically significant increases noted particularly in stages I and II. Notably, neutrophils in women afflicted with endometriosis may exhibit reduced responsiveness to weak activation signals. Nonetheless, as the disease progresses and more potent pro-inflammatory signals are generated, these cells appear to become activated, as documented by Riley et al. (83). The phagocytic function of neutrophils diminishes notably in women diagnosed with endometriosis; however, following surgical intervention, this capacity may revert to a physiological condition (84). Dietary interventions may modulate neutrophil function directly or through microbiota-derived metabolites. Evidence suggests that omega-3 polyunsaturated fatty acids (PUFAs), and vitamins C and E can influence neutrophil chemotaxis, oxidative activity, and antimicrobial responses (85–87).

Critical for the elimination of inappropriate cells, NK cells are cytotoxic innate lymphoid cells that secrete mediators, lysing transformed or infected cells and thereby constraining tumor proliferation and viral infections. The peritoneal fluid of women with endometriosis contains NK cells that exhibit decreased cytotoxic activity and display altered expression of activating and inhibitory receptors (88). This results in a their ability to identify and eliminate ectopic endometrial cells, thereby facilitating immune evasion. Dietary factors may modulate NK cell activity. Human intervention studies have demonstrated modulation of NK-cell activity following supplementation with long-chain n-3 PUFA, particularly eicosapentaenoic acid (EPA)/docosahexaenoic acid (DHA) (89), while emerging evidence suggests that vitamin C (90) and epigallocatechin gallate (EGCG) (91) may also influence NK cell functions.

DCs, which are essential for antigen presentation and cell activation, appear to adopt a tolerogenic phenotype in the context of endometriosis (92). An increase in the cell density of peritoneal DCs, along with alterations in the proportion of mature DCs relative to immature DCs, was observed in patients with endometriosis (93). Analyses conducted by Suszczyk et al. (94) demonstrated a higher percentage of myeloid DCs and plasmacytoid DCs expressing programed cell death ligand 1 (PD-L1) or programed cell death ligand 2 (PD-L2) in the peritoneal fluid compared to the plasma of endometriosis patients. Among dietary factors, the Mediterranean diet and its bioactive components, particularly n-3 PUFA and vitamin D, appear to exert substantial effects on DC activity, modulating their maturation, cytokine production, and antigen-presenting capacity (95, 96). **Table 1** presents data collected from publications over the past five years, demonstrating both quantitative and qualitative changes in immune cells in cases of endometriosis.

**Table 1:** Quantitative and qualitative alterations in immune cell populations associated with endometriosis.

Immune cell	Quantitative/qualitative changes	Biological material	Ref.
Macrophages	M2-to-M1 macrophage polarization reduced TGF-β1 production, fibrosis, and lesion progression	mouse peritoneal macrophages; mouse model; paired human eutopic and ectopic endometrial tissues	(97)
	macrophage functional activity depended on their origin: endometrial macrophages promoted lesion growth, whereas monocyte-derived macrophages limited lesion establishment	mouse peritoneal cavity, endometriotic lesions	(98)
	ectopic stromal cell-derived lactate induced M2 macrophage polarization *via* the METTL3/TRIB1/ERK/STAT3 pathway	human ectopic endometrial tissue, primary stromal cells, macrophages, mouse model	(99)
	endometriosis altered the abundance of large and small peritoneal macrophage populations; niclosamide restored macrophage homeostasis	mouse peritoneal fluid; endometriotic lesions	(100)
	increased abundance of CD68^+^ macrophages in the eutopic endometrium	human eutopic endometrial tissue	(101)
MCs	increased mast cell infiltration and activation in endometriotic lesions	human ectopic lesions, eutopic endometrium	(72)
	increased expression of HDC, HRH1-HRH4 and histamine-associated inflammatory genes	human transcriptomic dataset	(75)
	differences in tryptase-positive mast cell density among endometriosis subtypes	human ovarian endometriosis	(68)
	increased numbers of activated mast cells expressing tryptase and CPA3	human ovarian endometriosis	(69)
Neutrophils	reduced phagocytic activity, restored after surgery	human peripheral blood neutrophils	(84)
NK cells	reduced peripheral NK-cell cytotoxicity without changes in NK-cell numbers	human peripheral blood NK cells	(101)
	reduced expression of NKp46 and co-expression of activating receptors (NKG2C/NKp46 and NKG2D/NKp46), associated with impaired NK-cell cytotoxicity and increased IFN-γ production	human peritoneal fluid	(88)
DCs	increased proportions of myeloid DCs, and PD-L1^+^/PD-L2^+^ mDCs and pDCs, with elevated soluble PD-L1 and PD-L2 levels, suggesting activation of the PD-1/PD-L1/PD-L2 immune checkpoint pathway in endometriosis	human peripheral blood, peritoneal fluid	(94)
	increased peritoneal DC density with a higher proportion of immature DCs and reduced mature DCs; LPS-induced DC maturation reduced endometriotic lesion size	human peritoneal fluid; mouse peritoneal fluid; mouse model	(93)

### 3.2 Mediators

In addition to immune cell involvement, inflammatory mediators critically contribute to the pathophysiology of endometriosis. The production of cytokines, chemokines, and other factors during endometriosis is primarily attributed to activated peritoneal macrophages, recruited circulating monocytes, endometrial stromal cells, and endometriotic cells (102). Among the inflammatory mediators implicated in endometriosis, interleukin (IL)-1β, IL-6, tumor necrosis factor (TNF), C-X-C motif chemokine ligand 8 (CXCL8) and transforming growth factor-β (TGF-β) are the most extensively investigated. Although these cytokines are key mediators of inflammation, their contribution to endometriosis extends far beyond the regulation of inflammatory responses. Within the peritoneal microenvironment, they promote the establishment, survival, and progression of ectopic endometrial tissue by stimulating angiogenesis, enhancing cellular proliferation and tissue remodeling (103).

Elevated levels of mediators in the peritoneal fluid and, in many cases, in the serum of patients with endometriosis support their involvement in the inflammatory processes underlying disease progression (104–107). Women with endometriosis showed significantly elevated levels of both proIL-1β and IL-1β in peritoneal fluid compared with healthy controls. In addition, peritoneal macrophages collected from affected women secreted higher amounts of cytokines under both basal and stimulated conditions, suggesting an enhanced pro-inflammatory state that may contribute to the pathogenesis of endometriosis (108). The study by Taylor et al. (109) demonstrated that IL-1β accelerates the senescence of human endometrial stromal cells (ESCs) and impairs their decidualization capacity. IL-1β exposure promoted the acquisition of a senescence-associated secretory phenotype (SASP), characterized by increased secretion of pro-inflammatory factors, including IL-6, TNF, matrix metalloproteinase (MMP) 3, CCL2, CCL5, and CXCL8. These effects were further enhanced with increasing cell passage number, suggesting that IL-1β may amplify age-associated cellular senescence processes. The authors also showed that the effects of IL-1β are mediated through activation of the c-Jun N-terminal kinase (JNK) signaling pathway. IL-6 in the peritoneal fluid of women with endometriosis have been demonstrated to diminish NK cell cytolytic activity. Additionally, IL-6 has been shown to influence the quantity of NK cell cytolytic components *via* modulation of the Src homology 2 region containing protein tyrosine phosphatase-2 (110). IL-18 is another key pro-inflammatory cytokine implicated in the pathogenesis of endometriosis due to its role in regulating immune responses, inflammation, and angiogenesis. It contributes to the modulation of TNF and CXCL8 production, exhibits pro-angiogenic activity, and regulates the expression of intercellular adhesion molecule-1 (ICAM-1) through activation of the NF-κB signaling pathway, which may subsequently promote MMP expression and extracellular matrix remodeling (111, 112). Interestingly, elevated serum TNF levels have been associated with increased disease severity in women with endometriosis. In a study by Bedaiwy et al. (113), TNF concentrations in peritoneal fluid demonstrated high diagnostic accuracy for endometriosis, with 100% sensitivity and 89% specificity. Furthermore, soluble TNF receptors (sTNFR1 and sTNFR2) were significantly elevated in early-stage endometriosis but not in those with advanced disease, suggesting their potential utility as biomarkers for the detection of early disease.

Other pro-inflammatory cytokines have received comparatively less attention and remain poorly characterized in the context of endometriosis. Alterations in IL-2 levels have been reported in both blood and peritoneal fluid; however, the available findings are inconsistent. While some studies have demonstrated reduced IL-2 level in peritoneal fluid, particularly in patients with advanced-stage disease (114), others have reported increased IL-2 levels in infertile women with endometriosis (115). Similarly, several studies have reported elevated IL-15 levels in the peritoneal fluid and ectopic endometrial tissue of women with endometriosis (116, 117), although reduced concentrations have also been observed patients with advanced-stage disease (118). IL-15 may contribute to endometriosis progression by modulating immune responses and promoting angiogenic processes, including the proliferation of endometrial endothelial cells. Elevated IL-16 concentrations have been reported in the peritoneal fluid of women with advanced-stage endometriosis (stages III/IV). Moreover, stimulation of peripheral blood mononuclear cells with recombinant human IL-16 increased the production of IL-6, TNF, and IL-1β, suggesting that IL-16 contributes to the maintenance of the inflammatory peritoneal microenvironment (119). Additionally, Babah et al. (120) stated the rs4778889 polymorphism in the *IL16* gene promoter is associated with an increased risk of endometriosis in the Nigerian population, with the C allele identified as a potential genetic susceptibility factor.

Cytokines known for their anti-inflammatory and/or immunoregulatory functions, including IL-10 and TGF-β, also play important roles in the pathophysiology of endometriosis. Increased IL-10 concentrations have been reported in the serum of women with endometriosis, whereas elevated TGF-β levels have been detected in serum, peritoneal fluid, and endometrioma fluid. These findings are thought to reflect a compensatory response to chronic inflammation, aimed at limiting excessive tissue damage and maintaining immune homeostasis (121–124).

**Figure 2:**
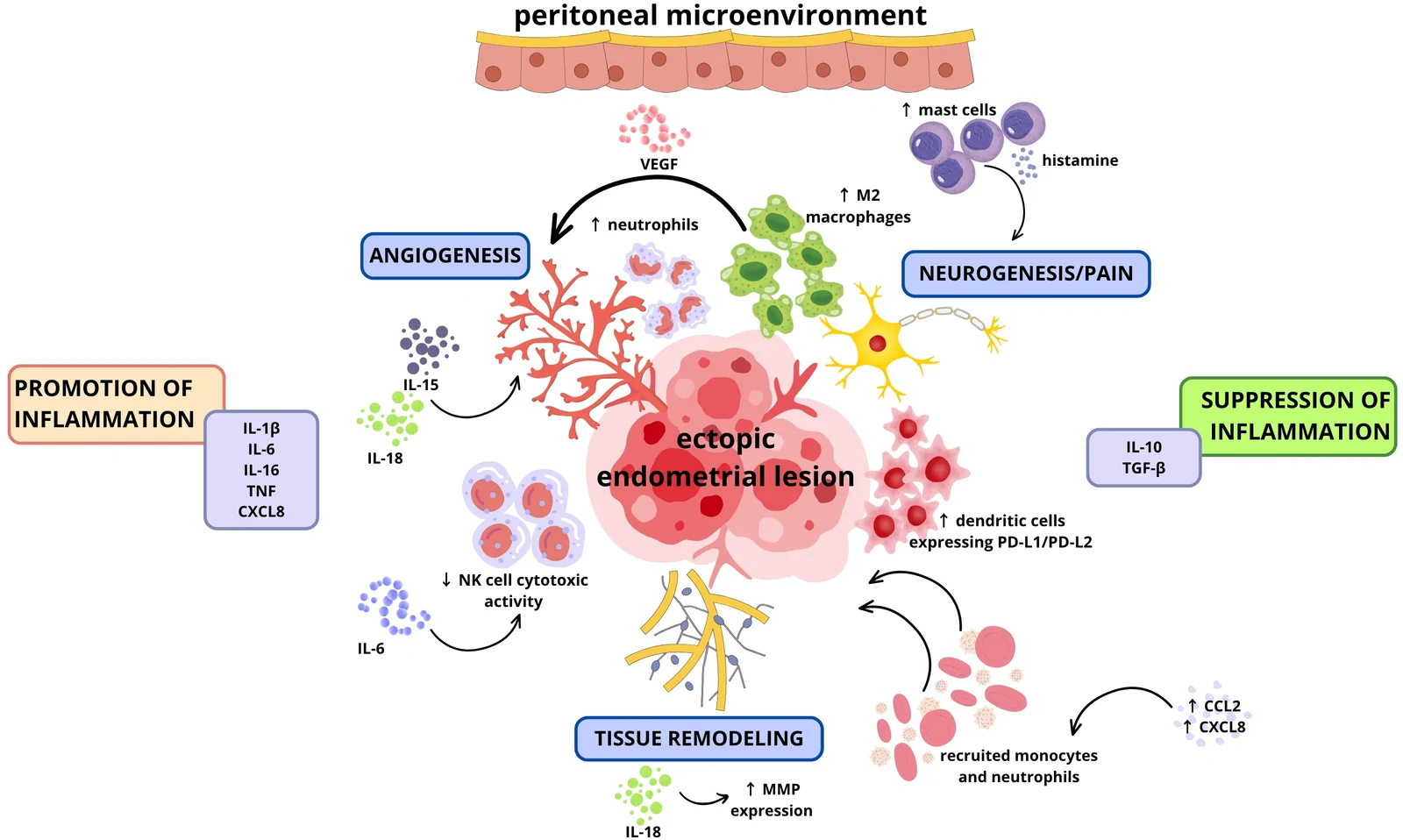
Immune dysregulation within the peritoneal microenvironment of endometriosis.

Elevated levels of chemokines, such as CCL2 and CXCL8, promote the recruitment of monocytes, macrophages, and neutrophils, with their concentrations increasing in parallel with disease severity (125). Concurrently, vascular endothelial growth factor (VEGF), produced by activated macrophages and endometrial cells in response to pro-inflammatory cytokines and estrogens, drives neovascularization, which is essential for lesion survival and growth (126). Overall, the immune microenvironment of endometriosis is characterized by a dynamic imbalance between persistent pro-inflammatory signaling and compensatory anti-inflammatory mechanisms, creating conditions that simultaneously sustain chronic inflammation while promoting immune tolerance and persistence of ectopic endometrial tissue. Dietary factors may also influence this inflammatory milieu by modulating systemic inflammatory and immune responses. In particular, healthy dietary patterns such as the Mediterranean diet have been associated with reductions in circulating inflammatory biomarkers, including C-reactive protein (CRP) and IL-6, suggesting that dietary modulation of inflammatory signaling may represent a potential mechanism relevant to endometriosis pathophysiology (127, 128). **Figure 2** depicts the major components and interactions involved in immune dysregulation within the peritoneal microenvironment of endometriosis.

Collectively, current evidence indicates significant immune dysregulation associated with endometriosis; however, its interpretation necessitates careful consideration of several limitations. Reported alterations differ among immune-cell populations, biological compartments, disease stages, and experimental models and do not necessarily reflect the local peritoneal or lesion microenvironment. Furthermore, quantitative changes in immune-cell abundance must be distinguished from modifications in phenotype or functional activity, as these facets may entail different biological implications. A substantial portion of the mechanistic evidence is obtained from experimental models, which constrains direct application to human pathology. Consequently, rather than representing a uniform inflammatory state, endometriosis seems to involve context-dependent immune remodeling characterized by altered inflammatory signaling, compromised immune surveillance, and compensatory immunoregulatory mechanisms.

### 3.3 Microbiota

**Figure 3:**
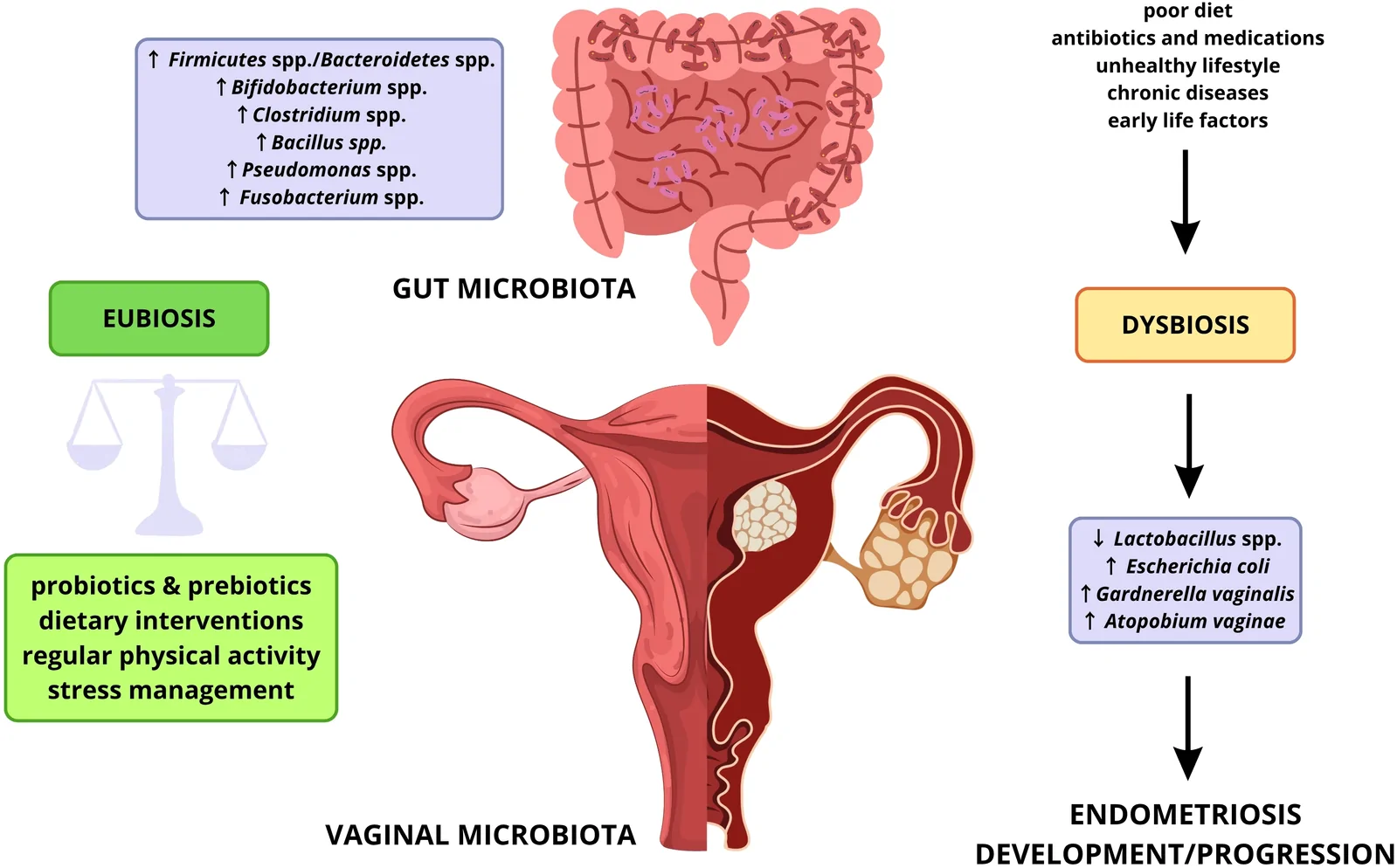
Microbiota-mediated mechanisms contributing to the development and/or progression of endometriosis.

The microbiota, which encompasses all microorganisms residing within the human body, undergoes variations in both quantity and quality throughout an individual’s lifespan. Factors such as genetics, lifestyle practices, dietary patterns – including the consumption of ultra-processed foods and food additives – and pharmacological treatments can induce disruptions in the composition or function of the microbiota. This disturbance, known as dysbiosis, manifests symptoms that depend on the affected site. Emerging evidence suggests a correlation between dysbiosis and various systemic diseases (129), notably endometriosis (130, 131). Studies indicate a bidirectional relationship among the gut, vaginal, and endometrial microbiota. Dysbiosis may create a pro-inflammatory environment that facilitates the ectopic tissue implantation and perpetuation of endometrial cells. Conversely, ongoing inflammation has the capacity to further alter the composition of the microbiota, thereby establishing a pathological feedback loop (132). **Figure 3** presents the microbiota-mediated mechanisms contributing to the development and/or progression of endometriosis.

Although the microbiota of the upper genital tract remains inadequately characterized, the vaginal microbiota has been extensively investigated. *Lactobacillus* species are acknowledged as pivotal contributors to a healthy vaginal environment through their role in maintaining an acidic pH and suppressing pathogen proliferation (133). Accordingly, the loss of *Lactobacillus* dominance may disrupt this protective environment and is linked to vaginal dysbiosis (133, 134). A vaginal microbiota characterized by a relative abundance of *Lactobacillus* spp. below 90%, has been associated with conditions such as bacterial vaginosis and other genital tract infections (133, 134). Such modifications in the microbial composition of the genital tract have also been observed in women suffering from endometriosis, including an increased prevalence of potentially pathogenic taxa such as *Escherichia coli*, *Gardnerella vaginalis*, and *Atopobium vaginae* (135–137). This transition to a less protective environment is associated with increased local inflammation, evidenced by elevated levels of MMPs, as well as higher concentrations of IL-6, TNF, and IL-1β (138). Gram-negative species that produce endotoxins, such as lipopolysaccharide (LPS), activate Toll-like receptor 4 (TLR4) signaling pathways, leading to increased production of inflammatory mediators that may exacerbate the development of ectopic lesions (139). Furthermore, this disturbance may contribute not only to the progression of endometriotic lesions but also to anxiety and depressive symptoms through microbiota – gut – brain axis signaling (140). Vaginal dysbiosis has been also associated with infertility, as it may adversely affect embryo implantation and endometrial receptivity (141). Some studies suggest that the restoration a *Lactobacillus*-dominated flora with probiotics or vaginal microbiota transplantation (VMT) could offer novel therapeutic strategies (142).

One of the earliest investigations into the relationship between endometriosis and the gut microbiota was carried out by Bailey and Coe (143). Employing a rhesus monkey model with spontaneous endometriosis, the authors identified an altered intestinal microbial profile characterized by a decreased abundance of *Lactobacillus* species, elevated levels of Gram-negative bacteria, and a heightened prevalence of intestinal inflammation relative to healthy controls. In murine models, endometriosis has been reported to modify the composition of the gut microbiome without significantly impacting overall microbiome diversity or richness (144). Following 42 days of endometriosis induction, mice demonstrated gut dysbiosis, marked by an increased *Firmicutes* to *Bacteroidetes* ratio and a higher abundance of *Bifidobacterium* compared to control specimens.

The rectal microbiota remains an emerging yet insufficiently explored aspect within endometriosis research (145). Given its anatomical proximity to DIE, the rectal environment may function as both a reservoir and a modulator of pelvic inflammation (146). Research conducted by Svensson et al. (147) indicates altered microbial profiles, including differences in the *Clostridia* and *Bacilli* classes, which are associated with inflammation and barrier disruption. Communication between the rectal and other gastrointestinal compartments may exacerbate immune dysregulation through shared lymphoid networks and microbial metabolites.

Recent studies have identified specific bacteria as potential novel contributors to the pathogenesis of endometriosis. *Fusobacterium* has emerged as a potential pathogenic factor, activating TGF-β signaling, promoting fibroblast differentiation, and enhancing endometriotic lesion formation (148). Similarly, Wu et al. (149) demonstrated that gut-derived *Pseudomonas* translocate to the peritoneal cavity, where it induces LPS-dependent NETosis by neutrophils, thereby promoting lesion development. In mouse models, targeting *Fusobacterium* or NETosis significantly reduced lesion growth, suggesting promising therapeutic targets for these pathways.

SCFAs produced by the gut microbiota constitute a potential connection between microbial metabolism and endometriosis. Due to their anti-inflammatory and immunomodulatory attributes, SCFAs may affect the inflammatory microenvironment related to the condition. Preclinical research indicates that microbiota-derived SCFAs, especially n-butyrate, could hinder the progression of endometriotic lesions; nevertheless, direct clinical evidence correlating SCFA levels with disease severity or pain remains sparse (150). In addition to local effects, dysbiosis may affect systemic symptoms associated with endometriosis. Dysregulation of the gut-brain axis has been linked to chronic pain, fatigue, and mood disorders (151). Changes in microbial metabolites including tryptophan catabolites (152), influence the central nervous system through vagal and immune-mediated pathways, potentially elucidating the high prevalence of psychological comorbidities observed in patients with endometriosis. The gut microbiota influences estrogen metabolism through the estrobolome - a collection of bacterial species capable of metabolizing estrogens (153). Dysbiosis may augment β-glucuronidase activity, resulting in elevated circulating estrogen levels and the stimulation of endometrial cell proliferation (154).

Both preclinical and clinical data support the utilization of probiotics, prebiotics, postbiotics, synbiotics, dietary modifications (155), and fecal microbiota transplantation (FMT) (156) to eliminate dysbiosis. Although these approaches show promise, most microbiota-targeted interventions for endometriosis are still in the experimental phase. Larger-scale clinical trials are required to determine their efficacy, safety, and the most effective delivery methods.

The immune mechanisms outlined above present several potential points of convergence with nutritional factors. An analysis of these immune and inflammatory pathways indicates that the most relevant targets for dietary intervention encompass macrophage-mediated cytokine signaling, eicosanoid metabolism, angiogenic pathways, and the gut microbiota-immune axis. Among these, macrophage activation and the secretion of pro-inflammatory cytokines, particularly TNF, IL-6, and IL-1β, are critical intervention points through which dietary constituents may influence the inflammatory environment associated with endometriosis. Eicosanoid metabolism constitutes a crucial aspect of the nutritional-immune interface, as the availability and composition of dietary fatty acids can influence arachidonic acid-derived signaling and the equilibrium of pro-inflammatory and anti-inflammatory lipid mediators through cyclooxygenase and lipoxygenase pathways. The complex interplay between inflammation and angiogenesis presents an additional potential target, specifically through VEGF and VEGF-C/VEGFR2 signaling pathways involved in lesion vascularization. Furthermore, the gut microbiota serves as a notably direct interface between dietary exposure and immune regulation, wherein modifications in microbial composition and metabolite production could impact immune cell activity and inflammatory signaling. Importantly, immune pathways in endometriosis are closely interconnected with estrogen signaling, establishing a potential hormone-immune-diet axis. Estrogen receptor signaling can influence the activation and phenotype of immune cells, including macrophages, whereas nutritional and metabolic factors may impact estrogen availability or metabolism through mechanisms involving dietary fiber, gut microbiota, the estrobolome, soy-derived isoflavones, and adiposity-related aromatase activity. This delineates an additional prospective pathway whereby nutritional factors may interact with estrogen-sensitive immune mechanisms. Collectively, these pathways delineate the primary mechanistic points at which nutritional factors may interact with immune dysregulation in endometriosis, thereby establishing a framework for comprehending the potential immunomodulatory effects of diet. The particular nutritional factors, their proposed mechanisms of action, and the experimental and clinical evidence linking them to these immune and inflammatory pathways are examined comprehensively in Section 4, “Role of Nutrition in Endometriosis”.

## 4 Role of nutrition in endometriosis

Dietary intake, including overall dietary patterns, nutrient composition, and the consumption of specific food components and bioactive compounds, can influence systemic inflammatory response (157), oxidative stress (158), and proper gut microbiota composition (159), all of which are involved in the pathophysiology of numerous human diseases. Emerging evidence indicates that dietary factors may substantially impact the development, progression, and severity of symptoms associated with endometriosis. Comprehending the interplay between nutrition and endometriosis may provide prospective pathways for supportive therapy and symptom control. Nutritional interventions are especially appealing owing to their non-invasive character, widespread availability, and capacity to enhance traditional treatment methods, thereby ultimately elevating patients’ quality of life.

### 4.1 Key nutrients and supplements

Various nutrients and bioactive dietary compounds have demonstrated potential relevance in the context of endometriosis owing to their anti-inflammatory, antioxidant, immunomodulatory, anti-angiogenic, and hormone-regulating properties. Among the most thoroughly researched nutrients are PUFAs, vitamin D, and a range of polyphenols, including resveratrol.

PUFAs, primarily obtained from fatty fish and flaxseed, demonstrate anti-inflammatory effects by substituting arachidonic acid (AA) as substrates for cyclooxygenase and lipoxygenase pathways. This displacement results in the reduction of pro-inflammatory eicosanoid biosynthesis (160). Interestingly, in a study involving 64 women with laparoscopically confirmed endometriosis and 74 healthy controls, the ratio of EPA to AA in serum was observed to exhibit a positive correlation with disease severity, suggesting that this ratio may serve as an indicator of endometriosis progression rather than merely its presence (161). These findings highlight that the comparative balance between pro-inflammatory and anti-inflammatory fatty acids, may play an important role in modulating the inflammatory processes involved in endometriosis pathophysiology and progression.

The efficacy of PUFA supplementation as an intervention for reducing endometriosis-associated pain in women has not yet been conclusively established. In a pilot, randomized, double-blind trial, PUFA supplementation was well tolerated and was not associated with any treatment-related adverse events; however, a significant reduction in endometriosis-related pain was not demonstrated (162). Similarly, a randomized, double-blind, placebo-controlled trial evaluated the effects of daily supplementation with 1000 mg of omega-3 fatty acids (fish oil) over a period of six months in young women aged 12 to 25 years diagnosed with surgically confirmed endometriosis and experiencing pelvic pain. Although a modest decrease in pain intensity, assessed using the Visual Analogue Scale (VAS), was observed in the omega-3 group, the difference did not reach statistical significance (163). In a retrospective observational study involving 289 women with endometriosis, the adjunctive supplementation of PUFAs at a median dose of 900 mg/day of EPA+DHA for 12 weeks was associated with more substantial reductions in pelvic pain, notable decreases in blood levels of inflammatory biomarkers (IL-6, TNF, and CRP), and enhancements in health-related quality of life, compared to traditional therapy alone (164). Nonetheless, due to the observational nature of the study, these results should be interpreted with caution and necessitate validation through well-structured prospective randomized controlled trials. A meta-analysis of five randomized controlled trials involving 424 women with endometriosis established that PUFA supplementation did not result in statistically significant improvements in pain intensity, sexual function, analgesic usage, pain catastrophizing, or quality of life when compared to control interventions. Nevertheless, PUFA supplementation was associated with a notable decrease in blood levels of inflammatory biomarkers, including pro-inflammatory cytokines such as TNF, IL-6, and IL-1, thereby indicating potential anti-inflammatory properties (165).

These mechanistic observations are corroborated by findings from experimental animal models of endometriosis. In a study by Covens et al. (166), using an *in vivo* New Zealand rabbit model of surgically induced endometriosis, supplementation with fish oil enriched with EPA and DHA markedly decreased the levels of PGE_2_ and PGF_2_α in the peritoneal fluid in comparison to the control group receiving olive oil. Moreover, eight weeks after endometriosis induction, the aggregate diameter of the endometrial implants was significantly reduced in the group supplemented with EPA/DHA relative to the control group. In a comparable investigation, Tomio et al. (167) demonstrated that fat-1 mice, which endogenously convert omega-6 fatty acids into omega-3 PUFAs, exhibited a lower number and smaller size of endometriotic lesions compared with wild-type controls. Lipidomic analysis indicated increased levels of EPA-derived metabolites, notably 12/15-HEPE, in fat-1 mice. EPA supplementation diminished lesion formation in wild-type mice; however, this effect was not observed in 12/15-LOX knockout mice, implying that the protective effects of EPA are mediated *via* the 12/15-LOX pathway and its metabolites. Furthermore, IL-6 expression within endometrial lesions was significantly reduced in fat-1 mice, indicating an attenuated local inflammatory response.

A summary of the available experimental evidence concerning other selected nutritional compounds and supplements in endometriosis is provided in **Table 2**. Randomized clinical trials have indicated that vitamin D and resveratrol may influence molecular pathways associated with endometriosis by decreasing the tissue expression of β-catenin, hs-CRP, VEGF, and TNF. Additionally, EGCG has demonstrated potent anti-angiogenic effects in experimental animal models through inhibition of the VEGF-C/VEGFR2 signaling pathway (168–172). Furthermore, probiotic supplementation with *Lactobacillus* has been shown to significantly alleviate dysmenorrhea and overall pain in women with surgically confirmed endometriosis. Although these findings are encouraging, larger-scale randomized clinical trials are required to verify their long-term efficacy and clinical significance.

**Table 2:** Key nutritional compounds and supplements in the management of endometriosis.

Nutrient/supplement	Source	Mechanisms	Evidence	Ref.
Omega-3 PUFAs (EPA/DHA)	fatty fish, fish oil, flaxseeds	anti-inflammatory activity; replacement of AA as cyclooxygenase/lipoxygenase substrate; reduced synthesis of pro-inflammatory eicosanoids; decreased inflammatory cytokines (TNF-α, IL-6, IL-1); modulation of 12/15-LOX pathway	meta-analysis of 5 randomized controlled trials (424 women): no significant improvement in pain intensity, sexual function, analgesic use or quality of life, but significant reduction in inflammatory biomarkers (TNF-α, IL-6, IL-1); observational study: 900 mg/day EPA+DHA for 12 weeks associated with reduced pelvic pain, lower IL-6, TNF-α and CRP, and improved quality of life; animal studies demonstrated reduced PGE_2_ and PGF_2_α levels, smaller endometriotic lesions and decreased IL-6 expression following EPA/DHA supplementation	(164–167)
Vitamin D	sunlight, fortified foods, supplements	regulation of the Wnt/β-catenin signaling pathway	randomized exploratory trial (34 women with stage III/IV endometriosis): 50.000 IU vitamin D_3_/week for 12–14 weeks reduced active β-catenin expression and the active/total β-catenin ratio in eutopic endometrium	(169)
		anti-inflammatory and antioxidant activity	randomized double-blind placebo-controlled trial (60 women with endometriosis): supplementation with 50,000 IU vitamin D every 2 weeks for 12 weeks significantly reduced hs-CRP, increased total antioxidant capacity (TAC), and improved pelvic pain	(172)
Resveratrol	grapes, berries	anti-inflammatory and anti-angiogenic activity; inhibition of VEGF and TNF-α expression	randomized exploratory trial (34 infertile women with stage III/IV endometriosis): supplementation with 400 mg/day resveratrol for 12–14 weeks significantly reduced VEGF and TNF-α gene and protein expression in the eutopic endometrium during the implantation window	(170)
EGCG	green tea	anti-angiogenic activity; inhibition of the VEGF-C/VEGFR2 signaling pathway	experimental endometriosis model (human endometrial tissue from 10 women transplanted into immunocompromised mice; 30 mice): EGCG 50 mg/kg/day administered intraperitoneally for 3 weeks inhibited angiogenesis by suppressing VEGF-C/VEGFR2 signaling and reduced endothelial proliferation, inflammatory signaling, and endometriotic lesion vascularization	(168)
Probiotics	fermented foods, supplements	modulation of host immune responses and gut microbiota	pilot randomized placebo-controlled trial (37 women with surgically and histologically confirmed stage III/IV endometriosis): oral LactoFem^®^ (one capsule daily) for 8 weeks significantly reduced dysmenorrhea and overall pain compared with placebo	(171)

### 4.2 Anti-inflammatory dietary patterns

The Mediterranean diet, a predominantly plant-based dietary pattern characterized by a high intake of vegetables, fruits, legumes, nuts, whole grains, and olive oil, moderate consumption of fish and dairy products, and limited intake of red and processed meat, is one of the most extensively studied and widely recommended dietary patterns for promoting overall health. A meta-analysis of randomized controlled trials by Keshani et al. (173) demonstrated that adherence to the Mediterranean diet can significantly reduce circulating inflammatory biomarkers, notably CRP, IL-6, and IL-17, thereby supporting its potential role in mitigating inflammation-related risks. Moreover, the Mediterranean diet has been associated with improved reproductive health outcomes in women (174), although research describing its impact on improving the quality of life of women with endometriosis remains limited. The evidence supporting the anti-inflammatory effects is primarily derived from broader populations, and the clinical significance of these effects for women with endometriosis remains uncertain.

Among common dietary patterns worldwide, the traditional Korean dietary practices, distinguished by a high consumption of fermented foods, fiber, and seafood, have been linked to anti-inflammatory effects (175) and may offer protective benefits against the onset and progression of endometriosis. Fermented foods, especially kimchi and fermented soy products, serve as abundant sources of probiotics that can aid in maintaining gut microbiota balance (176), enhancing intestinal barrier integrity (177), and decreasing systemic inflammation (178), thereby potentially mitigating symptoms associated with endometriosis. Currently, there is a paucity of data concerning dietary interventions that emulate the traditional Korean diet in women diagnosed with endometriosis. Nonetheless, relevant clinical evidence may offer supplementary context regarding non-conventional approaches employed in women with endometriosis. For example, a case report described a 32-year-old woman with recurrent endometriosis following multiple laparoscopic excisions and hormonal therapy (179). Following treatment with Traditional Korean Medicine, which included acupuncture, moxibustion, herbal medicine, and fumigation therapy, no recurrence was documented during the 34-month treatment and follow-up period. Furthermore, the patient experienced complete resolution of menstrual pain and a lengthening of her menstrual cycle from 24 days to a mean of 26.63 ± 2.28 days. While these findings imply a potential therapeutic advantage of Traditional Korean Medicine for recurrent endometriosis, they should be interpreted with caution owing to their basis in a single case report.

A summary of the available evidence from studies involving women with endometriosis, including the effects of the Mediterranean diet and Traditional Korean Medicine, as well as their proposed biological mechanisms related to endometriosis progression and symptom severity, is presented in **Table 3**.

**Table 3:** Proposed non-pharmacological interventions and their potential biological mechanisms in relation to the progression and symptoms of endometriosis.

Intervention	Key components	Main mechanisms	Impact	Ref.
Mediterranean diet	vegetables fruits whole grains legumes olive oil fish	modulation of inflammatory cytokines	reduced systemic inflammation	(173, 174)
Traditional korean medicine	acupuncture moxibustion herbal medicine fumigation therapy	not established	no recurrence during 34-month follow-up; reduced menstrual pain and prolonged menstrual cycle (single case)	(179)

Conversely, a high intake of red and processed meats and trans fatty acids products has been associated with an increased risk of endometriosis (180). Dietary patterns characterized by a high consumption of these pro-inflammatory foods may promote oxidative stress (181), chronic low-grade inflammation (182), and immune dysregulation (183). A high intake of animal fats may increase circulating estrogen concentrations, thereby contributing to the establishment and progression of endometriotic lesions (184). Furthermore, diets rich in high-glycemic-index foods and sugar-sweetened beverages have been implicated in activating NF-κB signaling (185), which may lead to increased production of pro-inflammatory cytokines, including IL-6 and TNF (186). Collectively, these molecular and inflammatory mechanisms may promote angiogenesis, cellular proliferation, and the survival of ectopic endometrial tissue, thereby facilitating disease progression.

### 4.3 Elimination and special diets

Australian researchers (187) reported that dietary modification is a frequently employed self-management strategy among women with endometriosis. The predominant approaches include reducing or eliminating gluten and dairy products, as well as adhering to a low-FODMAP diet. According to the authors, these dietary interventions are associated with moderate improvements in pelvic pain and the most significant benefits in gastrointestinal symptoms, along with reductions in nausea, vomiting, and fatigue. Despite the widespread adoption of dietary strategies, only a limited number of patients seek consultation with a dietitian (188). Furthermore, no single dietary approach has demonstrated superior efficacy, indicating that dietary treatments should be tailored to individual patient symptoms and preferences. **Table 4** provides an overview of the potentially harmful dietary components and their associations with endometriosis risk.

**Table 4:** Potentially detrimental dietary constituents and their impact on the risk of endometriosis.

Dietary components	Evidence-based mechanism	Impact	Study group (ref.)
Red and processed meats	higher intake may increase exposure to pro-inflammatory compounds and influence steroid hormone metabolism, contributing to a pro-inflammatory milieu	higher risk of laparoscopically confirmed endometriosis, especially with frequent red meat consumption	3800 women, USA (189)
Trans fatty acids	high intake may promote systemic inflammation by increasing pro-inflammatory cytokine production (e.g., TNF-α and IL-6) and endothelial dysfunction	higher risk of laparoscopically confirmed endometriosis	1199 women, USA (190)
		no consistent association with endometriosis risk	284 women, USA (191)
Saturated fats	high intake may enhance inflammatory signaling; however, direct evidence is inconsistent	no consistent association with endometriosis risk	1199 women, USA (190) 284 women, USA (191)
High-glycemic foods	high dietary glycemic index may influence postprandial glucose metabolism and systemic inflammatory responses	higher dietary glycemic index was associated with a modest increase in the risk of laparoscopically confirmed endometriosis; glycemic load showed no association	3810 women, USA (192)

Recent evidence suggests that a low-GI diet, originally devised for the treatment of irritable bowel syndrome (IBS), may further serve to mitigate gastrointestinal symptoms in women suffering from endometriosis, such as bloating, abdominal pain, and changes in bowel habits (193). By limiting the intake of fermentable oligosaccharides, disaccharides, monosaccharides, and polyols, this dietary strategy diminishes intestinal fermentation, gas production, luminal water content, and may reduce low-grade intestinal inflammation (194, 195). Although these findings are promising, the low-FODMAP diet should be undertaken under the supervision of a qualified healthcare professional, as extended adherence may elevate the risk of nutritional deficiencies and adversely affect the composition of gut microbiota (196).

In a study by Moore et al. (197), 72% of women diagnosed with both endometriosis and IBS reported more than a 50% improvement in bowel symptoms after four weeks of following a low-FODMAP diet. Based on the analysis of the available data, the pioneering comprehensive study on the low-FODMAP diet in endometriosis is credited to the Van Haaps study (198). The study included 62 women with endometriosis, of whom 22 followed a low-FODMAP diet, 21 followed an endometriosis diet, and 19 served as controls. After six months, women adhering to either dietary intervention reported significant reductions in pain in four of the six assessed pain symptoms compared with their baseline, as well as significant improvements in 6 of the 11 quality-of-life domains. When compared with the control group over the same follow-up period, the dietary intervention groups experienced significantly less bloating and demonstrated superior quality-of-life scores in 3 of the 11 assessed domains. These findings were validated in the study conducted by Keukens et al. (199). The authors of a study involving 24 patients with endometriosis discovered that adhering to a low-FODMAP diet was correlated with notable enhancements in constipation, endometriosis-associated pain, and various facets of women’s quality of life. Nearly two-thirds of participants reported a decrease in pain, especially chronic pelvic pain, and over fifty percent experienced relief from bloating. Furthermore, significant improvements were documented in emotional well-being, perceived control, social support, self-image, professional life, and sexual function. Similar conclusions were drawn in the research of Varney et al. (200); however, no changes were noted in the case of perceived stress, anxiety, or depression in women with diagnosed endometriosis.

The role of a gluten-free diet in endometriosis remains a topic of debate. Although some observational studies have documented improvements in pelvic pain, gastrointestinal symptoms, and quality of life following gluten elimination (201), these findings are derived primarily from uncontrolled or retrospective studies and should therefore be interpreted with caution. The proposed mechanisms suggest a reduction in gastrointestinal symptoms in susceptible individuals rather than a direct effect on the pathophysiology of endometriosis. Importantly, no high-quality randomized controlled trials have confirmed the efficacy of a gluten-free diet in women with endometriosis, and the existing evidence is inadequate to endorse its routine implementation (202). Additionally, long-term adherence to a gluten-free diet without medical indication may pose risks of insufficient intake of dietary fiber, B vitamins, and iron, and could adversely impact the gut microbiota if not properly supervised. Therefore, a gluten-free diet should be considered for women with diagnosed celiac disease or non-celiac gluten sensitivity rather than recommended as a routine therapeutic strategy for endometriosis.

Although plant-based diets have been proposed as a potential dietary approach to mitigate inflammation in women with endometriosis (203), no completed randomized controlled trials have yet evaluated their efficacy. Currently, ongoing studies should focus on assessing the impact of low-fat and whole-food, plant-based diets on pain severity, quality of life, and inflammatory biomarkers in this patient population. **Table 5** outlines the main dietary elimination strategies evaluated in endometriosis, which have been considered as potentially beneficial interventions.

**Table 5:** Dietary elimination strategies in endometriosis include low-FODMAP, gluten-free, and plant-based diets.

Intervention	Rationale	Evidence	Recommendation	Ref.
low-FODMAP diet	reduction of fermentable carbohydrates to alleviate gastrointestinal symptoms associated with endometriosis	single-blind randomized controlled crossover feeding trial (35 women with endometriosis and gastrointestinal symptoms): a 28-day low-FODMAP diet significantly reduced abdominal pain and bloating, improved stool consistency, and improved gastrointestinal- and endometriosis-related quality of life compared with a control diet	may be considered for women with persistent gastrointestinal symptoms under dietitian supervision	(200)
Gluten-free diet	proposed reduction of gastrointestinal and systemic inflammation	no high-quality randomized controlled trials in women with endometriosis; current evidence is insufficient to support routine use	recommended only for women with diagnosed celiac disease or non-celiac gluten sensitivity	(204)
Plant-based diet	higher intake of fiber, antioxidants and phytochemicals with potential anti-inflammatory effects	no published randomized intervention trials in women with endometriosis.	no evidence-based recommendation can currently be made	—

### 4.4 Other nutritional factors

In addition to the dietary components and specific dietary approaches discussed above, several other dietary factors may also contribute to the endometriosis. Although their mechanisms of action are less well understood, accumulating evidence suggests that they may influence disease progression by modulating inflammation, oxidative stress, hormonal balance, and immune function.

One of the dietary factors whose role in endometriosis remains under investigation is dietary fiber. On one hand, dietary fiber, particularly fermentable fractions such as inulin, fructooligosaccharides, and β-glucans, has become recognized as a significant regulator of gut microbiota composition and metabolic activity. Microbial fermentation of these fibers facilitates the production of SCFAs, which possess immunomodulatory and anti-inflammatory properties, maintain intestinal barrier integrity, and support immune homeostasis. Conversely, inadequate intake of dietary fiber has been linked to diminished SCFA production, increased intestinal permeability, and heightened systemic inflammation (204, 205). Additionally, dietary fiber may affect estrogen homeostasis by promoting fecal estrogen excretion and modulating the activity of the estrobolome, thereby potentially reducing enterohepatic estrogen recirculation (206).

Clinical evidence further substantiates a potential role of dietary fiber in the regulation of estrogen. In a study involving 62 premenopausal women, an increase in daily fiber intake from approximately 15 g to 30 g *via* wheat bran supplementation significantly decreased circulating concentrations of estrone and estradiol after two months, without influencing progesterone or sex hormone-binding globulin (SHBG) levels (207). These results were subsequently corroborated by Goldin et al. (208), thereby supporting the hypothesis that specific sources of dietary fiber may play a role in reducing circulating estrogen levels.

Conversely, epidemiological evidence remains inconsistent. In a cross-sectional analysis involving U.S. women aged 20–54 years, utilizing data from the National Health and Nutrition Examination Survey (NHANES 1999–2006), Chinese researchers reported a positive correlation between dietary fiber intake and the prevalence of endometriosis (209). Subsequent adjustment for potential confounding variables revealed that women within the highest quartile of fiber consumption demonstrated a significantly increased likelihood of having endometriosis compared to those in the lowest quartile. Nonetheless, the authors underscored that the cross-sectional design of the study limits causal inference, thereby emphasizing the necessity for well-constructed prospective studies to elucidate the association between dietary fiber intake and the risk of endometriosis.

Overall, the available evidence suggests that dietary fiber may beneficially influence several biological processes implicated in the pathogenesis of endometriosis, including gut microbiota composition, SCFAs production, intestinal barrier integrity, inflammatory responses, and estrogen metabolism. However, despite these plausible mechanistic benefits and supportive clinical findings, epidemiological studies have yielded inconsistent results. Dietary fiber includes diverse types, such as soluble and insoluble fibers, along with fermentable fibers like inulin, pectins, resistant starch, and β-glucans. The biological effects of these fibers may vary depending on their type, source, and fermentability. Consequently, current evidence is inadequate to establish a definitive correlation between dietary fiber intake and the risk or progression of endometriosis. Further well-designed prospective studies and randomized controlled trials are required to clarify the role of different types and sources of dietary fiber in the prevention and management of endometriosis.

Noteworthy observations have also been documented concerning indole-3-carbinol (I3C), a phytochemical inherently found in cruciferous vegetables. In a murine model of endometriosis, oral administration of I3C markedly impeded the vascularization and growth of endometriotic lesions without eliciting adverse anti-angiogenic or anti-proliferative effects on reproductive organs. These outcomes were accompanied by diminished proliferation of stromal and endothelial cells, as well as reduced expression of essential pro-angiogenic signaling molecules, including VEGFR2, PI3K, and phosphorylated ERK (pERK). Collectively, these findings imply that I3C may inhibit the establishment and progression of endometriotic lesions through targeting angiogenesis and cellular proliferation, thereby underscoring its potential as a prospective therapeutic agent for endometriosis (210).

In a prospective cohort study involving 3.829 incident cases of laparoscopically confirmed endometriosis, it was observed that higher soy consumption correlated with a diminished risk of the disease. Each additional weekly serving of soy was associated with an 8% reduction in the risk of endometriosis. Likewise, an increase in dietary isoflavone intake was inversely related to the risk of endometriosis up to approximately 4 mg/day, beyond which the protective effect plateaued. This inverse relationship was evident exclusively among women without concurrent infertility, indicating that the association between soy intake and endometriosis may vary according to infertility status (211).

Maintaining an appropriate body weight and reducing excess adipose tissue, especially visceral fat, may constitute a significant aspect of endometriosis management by diminishing both estrogen synthesis and systemic inflammatory burden. Adipose tissue, particularly visceral adipose tissue, functions as an active endocrine organ that expresses aromatase, thereby facilitating the conversion of androgens into estrogens. As a result, excess adiposity correlates with increased circulating estrogen levels and a state of chronic low-grade inflammation, both of which may collaboratively contribute to the onset and progression of endometriosis (212, 213).

In summary, these observations indicate the possibility of a hormone-immune-diet axis in endometriosis. Estrogen signaling is intricately connected with immune regulation within the endometriotic microenvironment and may affect the activity and phenotype of immune cells involved in inflammation and lesion persistence. Specifically, estrogen signaling *via* estrogen receptors has been linked to MC activation and the release of inflammatory mediators (72, 77), while estradiol-dependent macrophage responses may play a role in processes related to lesion innervation and pain (57). Furthermore, the altered macrophage phenotype observed in endometriotic lesions may contribute to angiogenesis, tissue remodeling, and lesion persistence (55, 56). Nutritional and metabolic factors that could influence estrogen availability or metabolism – including dietary fiber through its effects on the gut microbiota, the estrobolome, and enterohepatic estrogen circulation (206–208), soy-derived isoflavones (211), and adiposity through aromatase-mediated estrogen biosynthesis (212, 213) - may therefore intersect with estrogen-sensitive immune pathways. Nevertheless, direct evidence linking dietary modulation of estrogen metabolism to changes in immune cell function in women with endometriosis remains limited. Consequently, the hormone-immune-diet axis should currently be regarded as a plausible mechanistic framework rather than a definitively established causal pathway through which nutrition influences immune dysfunction in endometriosis.

### 4.5 Quality of evidence

The quality and extent of evidence underpinning nutritional interventions in endometriosis exhibit considerable variability across the different dietary strategies discussed within this review. Randomized controlled trials are available for certain interventions, including PUFA supplementation (162, 163, 165), vitamin D (169, 172), resveratrol (170), probiotics (171), and the low-FODMAP diet (200). Nevertheless, the study populations, intervention protocols, sample sizes, and clinical outcomes assessed vary among these interventions, thereby restricting direct comparisons and the broader applicability of the findings. In contrast, data concerning gluten-free and vegetarian diets remain limited, with no substantial randomized controlled trials presently advocating their routine use in women affected by endometriosis (201–203). Evidence relating to other nutritional factors is predominantly observational. For instance, associations between dietary fiber intake and endometriosis have yielded inconsistent results (206–209), whereas the link between soy/isoflavone consumption and endometriosis risk is supported by prospective cohort studies (211). Evidence concerning Traditional Korean Medicine, which is discussed separately from dietary interventions, is presently restricted to a single case report (179). Several proposed nutritional mechanisms are chiefly supported by preclinical evidence. For example, the potential anti-angiogenic effects of EGCG and I3C have primarily been demonstrated in experimental models (168, 210). Such mechanistic insights offer a biological rationale for further research but, in isolation, cannot establish clinical efficacy in women with endometriosis.

Overall, the available evidence encompasses randomized clinical trials, observational studies, case-based analyses, and exclusively preclinical research. This considerable heterogeneity in evidence quality and study methodologies should be considered when assessing the potential clinical significance of specific nutritional interventions. Currently, the evidence remains inadequate to endorse universal dietary guidelines for endometriosis, highlighting the need for meticulously designed, sufficiently powered, multicenter randomized controlled trials that incorporate standardized dietary protocols and clinically pertinent outcomes.

## 5 Recommendations and future perspectives

Accumulating evidence indicates that endometriosis is a multifactorial disease driven by complex interactions among genetic, immunological, hormonal, and environmental factors. These mechanisms collectively promote the establishment and persistence of ectopic endometrial tissue while sustaining the chronic inflammatory microenvironment characteristic of the disease. Although considerable progress has been made in elucidating the molecular basis of endometriosis, further studies are needed to translate these findings into clinically applicable biomarkers and targeted therapeutic strategies. Importantly, alongside genetic susceptibility, modifiable environmental factors, particularly diet, have emerged as potential regulators of inflammation, immune function, and estrogen metabolism. Consequently, growing attention has been directed toward the role of nutritional interventions as complementary strategies for the prevention and management of endometriosis.

Diet has emerged as a significant modifiable factor that can influence the pathophysiology and clinical manifestations of endometriosis through its effects on inflammation, immune function, oxidative stress, estrogen metabolism, and the gut microbiota. Consequently, dietary interventions have garnered increasing attention as complementary strategies for enhancing symptom management and quality of life in women with endometriosis. Based on the available evidence, dietary interventions should be regarded as a supplementary element of endometriosis management rather than a substitute for established medical or surgical therapies. Adherence to an anti-inflammatory dietary pattern, particularly the Mediterranean diet, combined with adequate intake of fiber, omega-3 polyunsaturated fatty acids, antioxidants, and vitamin D, may yield clinical benefits by modulating inflammation, oxidative stress, immune function, and estrogen metabolism. Maintaining a healthy body weight and reducing exposure to endocrine-disrupting chemicals may additionally support disease management. Furthermore, beyond individual nutrients and dietary patterns, metabolic and environmental factors may also play a role in disease progression. Therefore, although accumulating evidence indicates that nutritional interventions may influence key pathogenic pathways involved in endometriosis, additional well-designed randomized controlled trials are necessary to establish causal relationships and develop evidence-based dietary recommendations for clinical practice.

The future of endometriosis management is likely to extend beyond conventional symptom-oriented approaches toward precision medicine that integrates molecular, immunological, metabolic, and lifestyle-related factors. In this context, nutrition should no longer be viewed solely as supportive care but as a potentially modifiable environmental determinant capable of interacting with immune function, inflammatory signaling, oxidative stress, estrogen metabolism, and the gut microbiota. However, whether dietary interventions can modify disease progression or primarily improve symptom burden remains an open question.

Given the substantial clinical and biological heterogeneity of endometriosis, a universal dietary approach is unlikely to be effective for all patients. Future research should therefore focus on identifying immunological, metabolic, and microbiome signatures associated with distinct endometriosis phenotypes and differential responses to nutritional and conventional therapies. Integrating nutritional interventions with multi-omics technologies, immune profiling, and microbiome analysis may enable the development of personalized dietary strategies tailored to individual disease mechanisms. In this context, the concept of individualized nutrition is gaining increasing attention in the management of endometriosis, with particular emphasis on individual dietary requirements, food sensitivities, and patient-specific characteristics (214). Achieving this goal will require robust multicenter randomized controlled trials with standardized dietary protocols and comprehensive molecular characterization, ultimately facilitating the translation of mechanistic insights into evidence-based nutritional recommendations for women with endometriosis.
